# Functional MRI of Handwriting Tasks: A Study of Healthy Young Adults Interacting with a Novel Touch-Sensitive Tablet

**DOI:** 10.3389/fnhum.2018.00030

**Published:** 2018-02-13

**Authors:** Mahta Karimpoor, Nathan W. Churchill, Fred Tam, Corinne E. Fischer, Tom A. Schweizer, Simon J. Graham

**Affiliations:** ^1^Department of Medical Biophysics, Sunnybrook Research Institute, University of Toronto, Toronto, ON, Canada; ^2^Department of Neurosurgery, Keenan Research Centre of the Li Ka Shing Knowledge Institute, St. Michael’s Hospital, Toronto, ON, Canada; ^3^Geriatric Psychiatry, Department of Psychiatry, St. Michael’s Hospital, Toronto, ON, Canada

**Keywords:** handwriting, neuropsychological tests, fMRI, pen-and-paper test, visual feedback of hand position, ecological validity

## Abstract

Handwriting is a complex human activity that engages a blend of cognitive and visual motor skills. Current understanding of the neural correlates of handwriting has largely come from lesion studies of patients with impaired handwriting. Task-based fMRI studies would be useful to supplement this work. To address concerns over ecological validity, previously we developed a fMRI-compatible, computerized tablet system for writing and drawing including visual feedback of hand position and an augmented reality display. The purpose of the present work is to use the tablet system in proof-of-concept to characterize brain activity associated with clinically relevant handwriting tasks, originally developed to characterize handwriting impairments in Alzheimer’s disease patients. As a prelude to undertaking fMRI studies of patients, imaging was performed of twelve young healthy subjects who copied sentences, phone numbers, and grocery lists using the fMRI-compatible tablet. Activation maps for all handwriting tasks consisted of a distributed network of regions in reasonable agreement with previous studies of handwriting performance. In addition, differences in brain activity were observed between the test subcomponents consistent with different demands of neural processing for successful task performance, as identified by investigating three quantitative behavioral metrics (writing speed, stylus contact force and stylus in air time). This study provides baseline behavioral and brain activity results for fMRI studies that adopt this handwriting test to characterize patients with brain impairments.

## Introduction

Handwriting is a complex everyday skill that requires a combination of cognition, language processing, kinematics, motor planning, eye-hand coordination and visual-motor integration (Reisman, 1993; Rosenblum et al., 2003). Given this set of complex dependencies, it is not surprising that handwriting performance can be affected by many different brain impairments including Alzheimer’s disease (AD) (Platel et al., 1993; Croisile, 1999; Slavin et al., 1999; Schröter et al., 2003; Werner et al., 2006), developmental learning difficulties (Rosenblum et al., 2003), schizophrenia (Tigges et al., 2000), cerebrovascular disease (Auerbach and Alexander, 1981; Otsuki et al., 1999) and traumatic brain injury (Yorkston et al., 1997).

The functional neuroanatomy that supports handwriting performance has been investigated previously in multiple studies that have administered appropriate behavioral tests to patients with brain lesions. Whereas lesions of the angular gyrus produce impaired handwriting and impaired reading (Benson et al., 1982), other lesion studies have suggested the existence of handwriting-specific sites in parietal and frontal regions of the brain where lesions have been shown to affect handwriting but not other related tasks (Basso et al., 1978). Impaired handwriting but intact reading has been associated with lesions of the SPL (Yokota et al., 1990). Patients with lesions of the GMFA, a region of the MiFG close to the SFG, have shown impaired handwriting with preserved reading skills as well as intact simple motor functions (e.g., finger tapping) (Roux et al., 2009).

The exact contributions of regions such as the GMFA and the SPL in the control of handwriting remain incompletely understood (Planton et al., 2013). This uncertainty extends to the interpretation of neuropsychological (NP) tests developed to evaluate handwriting performance and associated brain impairments. It is generally known that the relationship between NP test scores and impaired brain function is complicated. The brain regions that support performance of specific NP tests may not have been fully and unambiguously identified, and a poor NP test score may arise from damage to one or more of the supporting interconnected brain regions (Lezak, 2004; Strauss et al., 2006). Therefore, it is difficult to localize the site of brain injury in patients with handwriting impairments using the appropriate NP tests. The limitations of using lesion studies to characterize the functional neuroanatomical correlates of a specific NP test are also well known (Hebben and Milberg, 2009).

An important approach to address these issues involves the use of task-based functional neuroimaging technology to probe the brain activity associated with handwriting. In particular, the fMRI method has been developed over approximately the last 25 years and is widely recognized as a safe, non-invasive and indirect probe of neural activity by neurovascular coupling mechanisms (Ogawa et al., 1990, 1992), at approximately millimeter spatial resolution throughout the entire brain.

Performing complex pen-and-paper tests during fMRI is challenging, however, from a logistical perspective. The fMRI methodology must be developed carefully to ensure that handwriting tasks are as ecologically valid as possible during imaging, recruiting similar aspects of brain function and behavior as handwriting administered under conventional testing conditions in the “real world.” The interplay between visuospatial, eye-hand coordination, and fine motor skills is particularly important in this context, and has led to methodological limitations in several previous studies. In two examples (Beeson et al., 2003; Segal and Petrides, 2012), subjects were instructed to write one word on top of the other using pen and paper without receiving visual feedback of their handwriting. Another study mapped brain activity while handwriting was performed with the index finger (Katanoda et al., 2001). Although the fMRI findings of such studies are partly consistent with the previous lesion work, they suffer from the limitation that the handwriting tasks were somewhat limited in their ecological validity.

For enhanced ecological validity of handwriting and drawing tasks, our lab previously designed and validated a computerized fMRI-compatible tablet system, consisting of a touch-sensitive surface that could be operated using a stylus (Tam et al., 2011). In an additional refinement, a video camera and augmented reality display were added to provide enhanced VFHP, which improved writing performance in young healthy adults (Karimpoor et al., 2015). Other studies have also shown that VFHP is an important human factor that should be included particularly for patients with brain impairments, as they may rely on this form of visuomotor integration to maintain performance on tasks that healthy individuals do not find challenging (Halligan et al., 1996). For example, AD patients show impaired performance when moving a cursor to a target, or when handwriting, when VFHP was not provided (Ghilardi et al., 1999, 2000; Slavin et al., 1999). Thus, providing VFHP may be advantageous to ensure that when patients with brain impairments are assessed using a specific NP test, the resultant test scores reflect impairment on the specific domains of interest attributed to the NP test, rather than confounds associated with difficulties in tablet interaction.

The purpose of the present work is to use fMRI methods together with the above-mentioned tablet technology to characterize the brain activity associated with an NP test developed specifically to assess handwriting performance and kinematics. This test was developed for use with a digitizing tablet to assess handwriting impairments (agraphia) in AD patients (Werner et al., 2006). Agraphia was observed in the original case study of AD (Maurer et al., 1997), and is recognized as a manifestation of the disease that includes both language processing difficulties as well as motor impairments such as reduced pen pressure and writing velocity (LaBarge et al., 1992; Croisile, 1999; Slavin et al., 1999). The NP test in question consists of three simple tasks: copying a grocery list; a phone number list; and a paragraph (sentence), that may require different levels of demand on handwriting behavior, language processing and brain activity. In the present study, the rationale for choosing this NP test was that (1) the initial work by Werner et al. (2006) indicated strong ability of this test to differentiate patients with mild cognitive impairment (MCI) and patients with early probable AD from healthy elderly controls; (2) this NP test has direct relevance to activities of daily living and especially day-to-day handwriting activities; (3) the functional neuroanatomical correlates of this NP test are not well determined; and (4) the test potentially provides a highly complementary adjunct to other NP tests typically applied to assess cognitive impairment in AD patients, such as the Mini-Mental State Examination (MMSE) (Folstein et al., 1975) and those that focus on memory (Blackwell et al., 2003). As a prelude to undertaking fMRI studies to investigate the performance of AD patients using this NP test, the present study consists of preliminary proof-of-concept validation work involving young healthy adults. It is hypothesized that the brain activity supporting this NP test (a) consists of a distributed network of regions in reasonable agreement with previous neuropsychological and neuroimaging studies of handwriting performance; and (b) shows differences between the test subcomponents that are consistent with different demands of neural processing for successful task performance.

## Materials and Methods

### Subjects

The study was conducted with the approval (# 080-2011) of the Research Ethics Board at Sunnybrook Health Sciences Centre in Toronto, and with the free and informed consent of the volunteer subjects. Twelve young, healthy adults (five male and seven female; mean age 25 years) were recruited from the population of graduate students at the University of Toronto. All subjects were right-handed as assessed by the Edinburgh Handedness Inventory (Oldfield, 1971); free from standard MRI exclusion criteria (e.g., claustrophobia, ferromagnetic implants); and free from any past or present neurological or psychiatric impairments.

### fMRI Tasks

**FIGURE 1:**
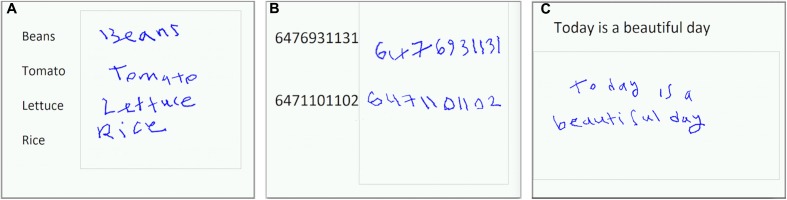
Handwriting tasks for fMRI and representative behavioral responses: **(A)** copying a grocery list; **(B)** copying phone numbers; **(C)** copying a sentence.

Three simple writing tasks were developed following an approach similar to that of Werner et al. (2006). The three tasks (**Figure 1**) consisted of (a) copying a grocery list; (b) copying phone numbers, and (c) copying a sentence. For the first two tasks, subjects were required to copy four grocery items and two phone numbers on one display screen, respectively. Visual stimuli were listed on the left side of the display screen, with a boxed area located on the right side for making handwriting responses. The procedure was similar for the third task, except that the response box was located below the sentence. Subjects were instructed to write in their usual writing style, in lowercase cursive script, as quickly as possible while maintaining accuracy. The maximum duration for each task was 35 s. It was anticipated that subjects would have variable completion times for each task block and that in some cases, the completion time would be less than the maximum duration. Subjects were therefore instructed to lift the stylus off the tablet surface once they had finished performing all elements of a given task, such that the stylus contact force data could be used to record task completion (see fMRI-compatible tablet technology, below). Each task was separated from the next by a baseline condition of visual fixation, consisting of a white screen with a central black fixation cross, of 10 s duration. Each task was repeated four times with different words and numbers for copying, and with the tasks presented in pseudo-random order. The fMRI time series data were subsequently collected in one “run” of 9 min and 10 s duration, containing all three handwriting tasks and baseline conditions. The time series also included an initial 10 s of dead time to ensure that the fMRI signal was at equilibrium prior to presenting the first handwriting task block.

Outside the magnet prior to fMRI, subjects practiced one trial of each writing task to become familiar with using the tablet. The practice trials involved novel stimuli that were not repeated during imaging.

### fMRI-Compatible Tablet Technology

Subjects operated the tablet while lying supine in the magnet bore, with the tablet mounted on an adjustable support stand that lifted the touch-sensitive surface away from the torso. The support ensured that respiratory motion did not move the tablet and thereby affect task performance. The position and angle of the support were also adjusted for each subject to enable tablet interactions comfortably with forearm or wrist support. Foam padding was used to eliminate possible discomfort at pressure points, such as the elbow in contact with the magnet bore. The tablet system included the same resistive touch-sensitive surface used in previous fMRI studies for converting localized contact force to position coordinate values, and for locating these values on a computer display (Tam et al., 2011). A force sensor (FSR 400, 30-49649, Interlink Electronics, Camarillo, CA, United States) was also located at the stylus tip to record relative force values as a function of time. Both (*x*, *y*) coordinates and force parameters were sampled and logged to a computer file at a rate of approximately 40 Hz using E-prime software (Psychology Software Tools, Inc., Sharpsburg, PA, United States).

The tablet was also equipped with an MRI-compatible video camera with a color CMOS sensor (12M-i, MRC Instruments Gmbh, Germany) and custom illuminator (Karimpoor et al., 2015). Using custom programs written in MATLAB (The Mathworks Inc., Natick, MA, United States) a real-time segmentation procedure was used to isolate the hand and stylus from each camera video frame, using a simple skin color detection algorithm performed in Red–Green–Blue (RGB) space (Kovac et al., 2003). In addition, the color properties of the stylus were adjusted to fall within the RGB distribution of skin color, using a red plastic cover. Touching the stylus to the tablet and pressing downward beyond a preconfigured force threshold resulted in “ink” marks at the appropriate locations on the display. The camera and stimulus/response video signals were then superimposed in an augmented reality display, projected on a rear-projection screen and viewed by the subject via a mirror on the MRI system’s head coil.

### Behavioral Data Analysis

Digitized tablet recordings were processed for each task and for each subject to extract three metrics similar to those reported by Werner et al. (2006): mean handwriting speed (pixels/s); the mean time that the stylus was held in the air during handwriting; and mean stylus contact force. Writing speed, s, was quantified by dividing the number of pixels “inked” during a given block, by the performance time (the completion time or the block duration, as appropriate). The time that the stylus was held in the air (in air time, IAT) during writing performance was quantified as the total time that zero force was recorded during each task. The stylus contact force, F, was quantified as the temporal average of the force recording data for each task. Mean values were determined for each metric by averaging over all trials of each task. The data obtained for each metric were also submitted to one-sample Kolmogorov–Smirnov tests to assess for normal distributions. Statistically significant differences in each metric were subsequently assessed between tasks using the Friedman test in MATLAB (The Mathworks Inc., Natick, MA, United States), with a Bonferroni *post hoc* correction for multiple comparisons (three comparisons; corrected alpha value = 0.017).

### Imaging Parameters

All imaging was conducted at 3.0 T using a research-dedicated whole-body MRI system (MR750, GE Healthcare, Waukesha, WI, United States), with a standard 8-channel head coil receiver. An angled mirror was attached to the head coil so that the subject could view visual stimuli on a rear-projection screen mounted at the rear of the magnet bore. Anatomical MRI was undertaken using inversion recovery-prepped three dimensional (3D) fast spoiled gradient echo imaging [3DFSPGR, inversion time (TI) = 300 ms, repetition time (TR) = 7.0 ms, echo time (TE) = 3.1 ms, flip angle = 15°, FOV = 22 cm × 22 cm, matrix = 256 × 192, number of slices = 190, slice thickness = 1 mm]. These images subsequently served as an anatomical underlay to the color maps of brain activity generated from the fMRI data. Functional MRI was undertaken using a T2^∗^-weighted spiral in/out pulse sequence (Glover and Law, 2001) to record brain activity via the BOLD effect (Ogawa et al., 1990, 1992) (TR = 2 s, TE = 30 ms, flip angle = 70°, FOV = 20 cm × 20 cm, matrix = 64 × 64, number of slices = 30, slice thickness = 4.5 mm). Cardiac and respiratory signals were measured during fMRI using a photoplethysmograph attached to the finger of the left hand and a respiratory belt strapped around the torso, respectively.

### fMRI Analysis

Previously, it has been demonstrated that the choices made in fMRI pre-processing pipelines (the procedures conducted to remove noise sources from fMRI data prior to estimating brain activity) significantly affect the reliability of the end results at both the individual and group level (Churchill et al., 2012a,b, 2015). This is especially the case when dealing with weaker contrasts, such as the differences in brain activity that are anticipated between the handwriting tasks in the present study. Furthermore, the present study provides preliminary, proof-of-concept results for a small sample size of volunteers. The Optimization of Preprocessing Pipelines for NeuroImaging (OPPNI) methodology was chosen for fMRI analysis, therefore, specifically for its ability to provide robust results while suppressing bias from subject-dependent noise sources – achieving improved signal detection and reliability of brain activations compared to pre-processing use of fixed pipelines for groups of subjects (Churchill et al., 2012a,b, 2015). Preprocessing pipelines were optimized for each single subject (prior to group analysis) based on metrics of prediction and reproducibility, generating the most reliable and task-predictive activation maps using the Non-parametric Prediction, Activation, Influence, and Reproducibility reSampling (NPAIRS) method (Strother et al., 2002) as briefly described below.

For each subject, the fMRI time series data were divided into two “split-halves,” corresponding to the first and second halves of the data in temporal order. The first two time points from each instance of each handwriting task and each baseline condition were discarded to avoid fMRI signal transients and to model stabilized BOLD hemodynamic responses. The fastest time for a subject to complete one writing task was 14 s, and thus only the first 14 s of each handwriting task was subsequently analyzed for all subjects. This choice simplified the fMRI analysis because it ensured that all subjects were engaged in task performance during the time designated in the analysis model for the task condition, removing the need to model effects of subject-specific completion time across the allotted 35 s time window for tablet interactions. The optimal preprocessing pipelines were selected by computing metrics of prediction (P) and reproducibility (R) for all preprocessing pipelines, and then selecting the pipeline minimizing the Euclidean distance D (P, R) relative to perfect model performance (*P* = 1, *R* = 1). The *P*-values were computed using a classifier model based on a single split-half, and measuring the ability to predict experimental conditions in the other split-half using Bayesian posterior probability from a LDA. The *R*-values were computed from the Pearson correlation between split-half activation maps. Pipeline optimization was conducted for each subject, by measuring D (P, R) for all possible combinations of the following preprocessing algorithms, implemented using calls to Analysis of Functional NeuroImages freeware (AFNI) (Cox, 1996) or directly in the OPPNI software. These algorithms were applied in the following fixed order: rigid-body motion correction using *3dvolreg*; cardiac and respiratory physiological noise correction (Glover et al., 2000) using *3dretroicor*; slice timing correction using *3dTshift*; spatial smoothing using a 6 mm FWHM Gaussian filter using *3dmerge*; temporal detrending with zero to third order Legendre polynomials using *3dDetrend*; motion covariate regression using PCA of *3dvolreg* motion parameter estimates and regressing principal components (PCs) that accounted for >85% of motion variance; task paradigm covariate regression to protect against over-regression of task-related BOLD signals, after convolving the paradigm with the AFNI ‘SPMG1’ function (Rasmussen et al., 2012); and global signal regression by removing the first component of PCA (Carbonell et al., 2011). Pipeline optimization was conducted by testing the impact of including/excluding all of the above pipeline steps except spatial smoothing, and selecting the pipeline minimizing D (P, R) for each subject. Brain masks were generated using the Oxford Centre for Functional MRI of the Brain (FMRIB) Software Library (FSL) Brain Extraction Tool (Smith et al., 2004).

The individual subject datasets were analyzed to obtain (P, R) values and SPMs using LDA, a predictive multivariate model, regularized by projecting onto a PCA basis prior to analysis [where the optimal number of PCs was chosen to minimize D (P, R)]. The LDA–PCA algorithm was chosen as a simple, robust multivariate classifier model, enabling computation of (P, R) metrics for each pipeline. Recent work has shown that compared to other multivariate linear classifiers, LDA–PCA exhibits relatively high prediction accuracy and highly reproducible activation maps (Churchill et al., 2014; Yourganov et al., 2014), of particular importance in the low sample size scenario. For each subject, the resulting brain maps (generated by LDA–PCA) were then converted into reproducible *Z*-scored statistical parametric maps (rSPMs) using the procedure described in (Strother et al., 2002) prior to group-level analysis. Subjects with low reproducibility have low rSPM *Z*-scores, such that they contribute less to the most reproducible activation pattern for the overall group-level analysis (Strother et al., 2002). The rSPMs were subsequently transformed into a standard brain atlas space (MNI 152, Mazziotta et al., 2001) as follows: FSL *flirt* was used to compute the rigid-body (6-parameter) transform from fMRI images to T1-weighted images, and the affine (12-parameter) transform from T1-weighted images to the MNI template. The net transformation matrix from fMRI images to the MNI template was then computed, and applied to the rSPMs.

Group-level analysis was then performed by applying PCA to the set of individual subject rSPMs, and extracting the spatial PCs that explained the most data covariance. A split-half cross-validation framework was used at this point in the analysis as in Churchill et al. (2015) with 100 resampling iterations, to obtain a *Z*-scored map of brain regions associated with each PC. This procedure is very stable, such that only a small number of resampling iterations is required for robust parameter estimation (Churchill et al., 2013). Previous studies have used as few as 50 iterations (Strother et al., 2004). The contrasts that were subsequently investigated included (a) all handwriting tasks vs. baseline, identifying areas of common brain activity across the tasks; (b) copying paragraphs vs. phone numbers; and (c) copying paragraphs vs. grocery lists (results for the contrast of copying phone numbers vs. the grocery list were not statistically significant). Each contrast was optimized separately using OPPNI to address the potential concern that there might be different optimized pipeline steps required for each subject between two different contrasts to achieve maximum signal detection and reproducibility of the output activation maps. However, the resampling framework used in OPPNI is not presently able to account for repeated measures multivariate analysis of variance. The maps of brain activity for each contrast were thresholded using the two-tailed False-Discovery Rate (FDR) to correct for multiple comparisons (Genovese et al., 2002), rather than using a family-wise error (FWE) approach with a cluster-size threshold (Eklund et al., 2016). The FDR method is well established and although it provides somewhat weaker control over type I error than FWE methods (Nichols and Hayasaka, 2003), it is simple to implement and does not require an estimation of the likelihood of a given cluster size occurring by chance. Given the preliminary nature of the study, activation maps were reported at a conservative FDR of *q* = 0.01.

## Results

**FIGURE 2:**
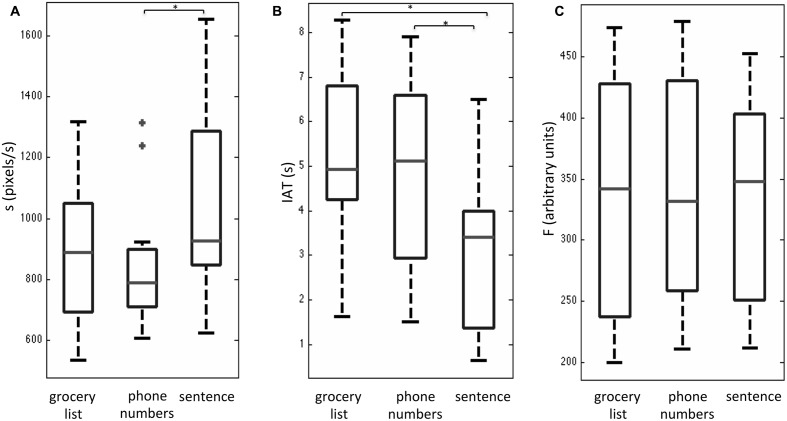
Box and whisker plots of behavioral performance metrics **(A)** handwriting speed s, **(B)** stylus in air time, IAT, and **(C)** stylus contact force, F for three handwriting tasks during fMRI, involving copying a grocery list, phone numbers, and a sentence. For each box, the interior horizontal line shows the median value, and edges of the box are estimates of the first and third quartile. The whiskers extend to the most extreme data points not considered outliers (2.7 times the sample standard deviation assuming a normal distribution). Outlier points are shown as crosses. Statistically significant differences between tasks (*p* < 0.05) are indicated by an asterisk.

After a brief training session outside the MRI system, all subjects reported that they were able to interact with the tablet easily during fMRI. Overall, subjects had completion times (group mean with standard deviation shown in brackets) of 23.1 (4.6) s for the grocery list task, 23.0 (5.4) s for the phone number task, and 26.2 (4.1) s for the sentence task. No statistically significant differences were observed between these completion times based on a one-way Friedman test with a Bonferroni correction for multiple comparisons. The behavioral results that were obtained for the three writing tasks during fMRI are shown as box and whisker plots in **Figure 2**. These plots show the median and interquartile range (IQR) for each metric (s, IAT, F) with the box bounding the first and third quartiles, and the whiskers extending to the most extreme data points not considered outliers (2.7 times the sample standard deviation). Outlier data points are shown as crosses. The sentence task showed significantly elevated *s*-values (**Figure 2A**) and reduced IAT values (**Figure 2B**) compared to the phone number task (Bonferroni corrected alpha value = 0.017; *p* < 0.01). In addition, the sentence task had a non-significant trend of increased *s*-values (*p* < 0.06), and significantly reduced IAT values (*p* < 0.002) compared to the grocery list task (**Figures 2A,B**). No statistically significant differences in *s* and IAT values were observed between the phone number and grocery list tasks. Furthermore, no statistically significant results were observed in the *F*-values across the three handwriting tasks (**Figure 2C**).

**FIGURE 3:**
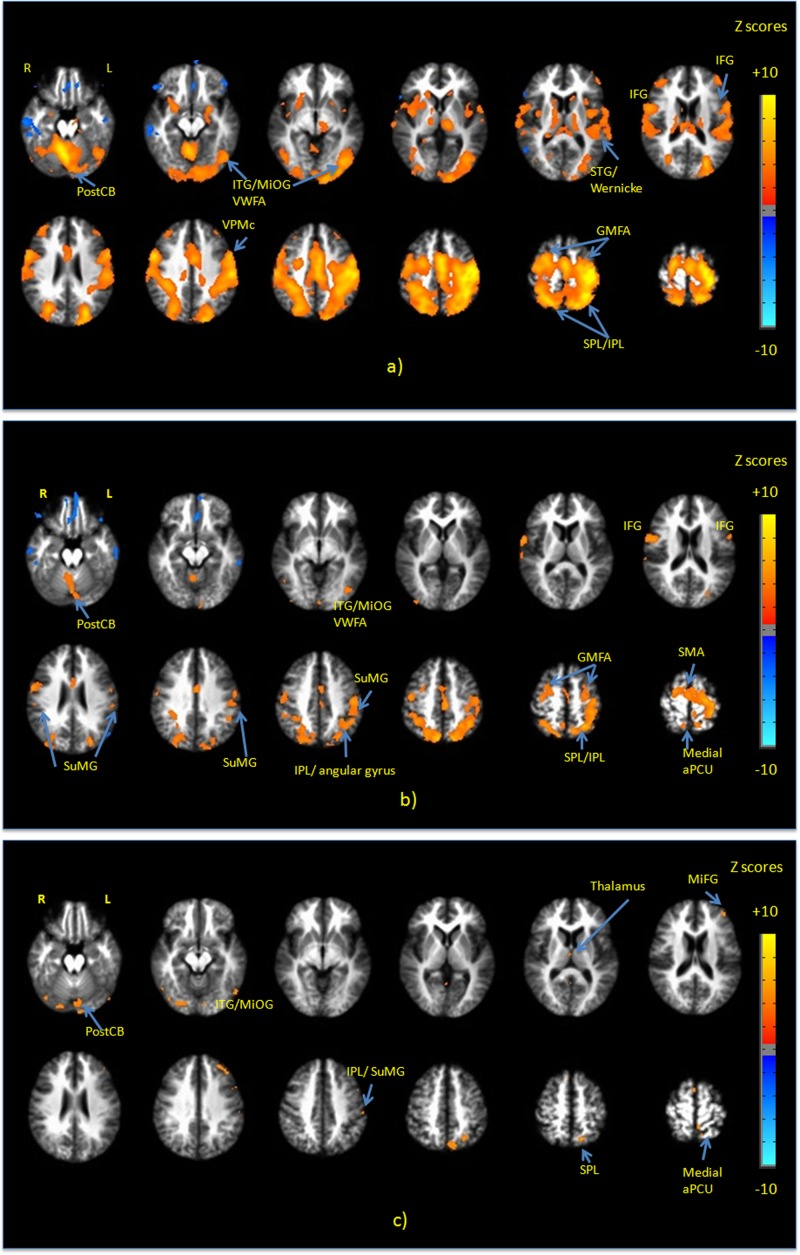
Activation maps of the first principal component (PC1) for **(a)** all handwriting tasks vs. baseline; **(b)** copying a sentence vs. copying phone numbers; and **(c)** copying a sentence vs. copying a grocery list. All activations are thresholded at the false discovery rate of *q* = 0.01. Activation maps were not statistically significant for any other principal components. IFG, inferior frontal gyrus; MiFG, middle frontal gyrus; STG, superior temporal gyrus; ITG, inferior temporal gyrus; MiOG, middle occipital gyrus; IPL, inferior parietal lobule; SPL, superior parietal lobule; SMA, supplementary motor area; PostCB, posterior cerebellum; VWFA, visual word form area; aPCU, anterior precuneus; GMFA, graphomotor frontal area; SuMG, supramarginal gyrus.

**Figure 3a** shows group maps of selected slice locations for the first principal component (PC1), reflecting the most common pattern of brain activity for all writing tasks vs. baseline. The associated complete set of regions activated is listed in **Table 1**. Results for all other PCs were negligible. Activations (not all shown in **Figure 3**) included the left-lateralized primary somatosensory and motor cortex, as well as the bilateral SMA and pre-motor, primary visual and visual association areas. In addition, activity involved the bilateral SPL, IPL, MiFG/SFG, STG, Wernicke’s area, postCB, angular gyrus, SuMG, bilateral IFG/Broca’s area, as well as ventral pre-motor cortex, and the left lateralized VWFA located in ITS/MiOG.

**Table 1:** Activated brain regions identified for all handwriting tasks vs. baseline in MNI coordinate space.

Active region	Hemisphere	*Z*-score	Uncorrected *P*-value	MNI (mm)	Coordinates	
Precentral gyrus	L	13.7	1.0e-42	-34	-28	66
SPL/IPL	L	10.6	3.0e-26	-36	-52	58
SPL/IPL	R	6.4	1.6e-10	34	-44	56
postCB	R	10.1	5.5e-24	6	-64	-20
SMA	L	9.4	5.5e-21	-4	-14	50
SPL	R	9	2.3e-19	22	-62	64
Precentral gyrus/Premotor	L	7.8	6.2e-15	-56	-6	40
MiOG/(SOG)	L	8.9	5.6e-19	-28	-78	24
IFG	R	9.3	1.4e-20	56	4	34
IFG	L	5	5.7e-7	-58	10	22
Insula	R	4.7	2.6e-6	40	-2	2
Insula	L	3.9	9.6e-5	-44	-6	6
Putman	R	5	5.7e-7	24	4	-1
Putman	L	3.1	1.9e-3	-24	12	-2
Caudate	R	4.5	6.8e-6	14	-2	14
MiOG/V5 (visual area)	L	7.5	6.4e-14	-40	-78	0
Precentral gyrus/premotor	R	7.7	1.4e-14	42	-8	56
Thalamus	L	5.5	3.8e-8	-12	-20	4
Thalamus	R	4	6.3e-5	12	-16	6
SFG/MiFG (GMFA)	R	7.8	6.2e-15	20	-6	68
MiFG/SFG (GMFA)	L	6.9	5.2e-12	-22	-10	56
Angular gyrus/IPL	R	7.5	6.4e-14	32	-42	40
Angular gyrus	L	4.5	6.8e-6	-36	-66	44
SuMG	L	4.4	1.0e-5	-52	-36	30
SuMG	R	3.6	3.2e-4	48	-32	36
Posterior STG/Wernicke	L	4	6.3e-5	-68	-36	14
STGAVernicke	R	3.6	3.2e-4	60	-32	14
Parahippocampus	L	4.6	4.2e-6	-26	2	-12
Parahippocampus	R	3.2	1.4e-3	22	-0	-14
Hippocampus	L	3.3	9.7e-4	-26	-14	-12
Ventral premotor (VPMc)	L	6.2	5.6e-10	-58	8	26
IOG/visual	R	4.8	1.6-e-6	30	-86	-14
Cuneus/visual	L	6.3	3.0e-10	-20	-96	-4
MiOG/ITG (VWFA)	L	4.5	6.8e-6	-46	-62	-12
MeFG	L	-5.3	1.2e-7	-4	36	-16
MiTG	L	-4.9	9.6e-7	60	-22	-16
IFG	R	-4.7	2.6e-6	52	36	-14
IFG	L	-4.2	2.7e-5	-50	40	-14

*IFG, inferior frontal gyrus; MiFG, middle frontal gyrus; MeFG, medial frontal gyrus; SFG, superior frontal gyrus; STG, superior temporal gyrus; ITG, inferior temporal gyrus; MiOG, middle occipital gyrus; SOG, superior occipital gyrus; IPL, inferior parietal lobule; SPL, superior parietal lobule; SMA, supplementary motor area; SuMG, supramarginal gyrus; VPMc, ventral premotor cortex; VWFA, visual word form area; MNI, Montreal Neurological Institute*.

**Figure 3b** shows PC1 for sentence vs. phone number tasks, with the complete list of differentially activated regions given in **Table 2**. Activity involved the bilateral SPL/IPL, pre-cuneus, IFG, SMA, premotor, MiFG/SFG (GMFA), precentral gyrus, and IPL/SuMG, as well as left lateralized somatosensory and primary motor cortex, ITG/MiOG (VWFA), and angular gyrus. Right-lateralized activity was also observed in the postCB.

**Table 2:** Activated brain regions identified for copying a sentence vs. copying phone numbers in MNI coordinate space.

Active region	Hemisphere	*Z*-score	Uncorrected *p*-values	MNI Coordinates (mm)		
SPL/IPL	L	7.2	6.0e-13	-28	-58	60
SPL/IPL	R	5.2	2.0e-7	26	-56	60
Precuneus	R	7.5	6.4e-14	8	-74	52
Precuneus	L	5.3	1.2e-7	-24	-74	52
Precentral gyrus (primary somatosensory cortex)	L	6.6	4.1e-11	-24	-18	68
Precentral gyrus	R	4.6	4.2e-6	34	-16	60
SMA	L	4.1	4.1e-5	-4	-4	64
PostCB	R	5	5.7e-7	6	-56	-16
MiFG/SFG (GMFA)	L	3.8	1.4e-4	-26	-10	56
MiFG/SFG (GMFA)	R	3.7	2.2e-4	26	-0	54
IFG	R	5.6	2.1e-8	52	2	22
IFG	L	3.8	1.4e-4	-60	6	20
Angular gyrus	L	4.7	2.6e-6	-40	-56	44
SuMG/IPL	L	3.6	3.2e-4	-58	-24	32
SuMG/IPL	R	3.5	4.7e-4	56	-24	26
MiOG/ITG = VWFA	L	4.8	1.6e-6	-42	-74	-6
Medial anterior precuneus	R	4.4	l.le-5	4	-54	62
(aPCU)						
Medial aPCU	L	4.3	1.7e-5	-12	-54	64
Lingual gyrus (visual)	L	4	6.3e-5	-0	-92	-4
MeFG	R	-5.2	2.0e-7	-2	32	-14
IFG	R	-4.7	2.6e-6	50	36	-18
MiTG	L	-4.1	4.1e-5	-64	-14	-18
ITG	R	-4	6.3e-5	64	-16	-28
Parahippocampal gyrus	R	-3.4	6.7e-4	34	-20	-22

*IFG, inferior frontal gyrus; MiFG, middle frontal gyrus; MeFG, medial frontal gyrus; SFG, superior frontal gyrus; STG, superior temporal gyrus; ITG, inferior temporal gyrus; MiOG, middle occipital gyrus; IPL, inferior parietal lobule; SPL, superior parietal lobule; SMA, supplementary motor area; PostCB, posterior cerebellum; VWFA, visual word form area; aPCU, anterior precuneus; GMFA, graphomotor frontal area; SuMG, supramarginal gyrus; MNI, Montreal Neurological Institute*.

**Figure 3c** shows PC1 for sentence vs. grocery list tasks, with all activated regions listed in **Table 3**. Activity involved the bilateral visual areas and lingual gyrus as well as ITG/MioG (VWFA), left lateralized SPL, IFG, insula, SuMG, post-central gyrus and the medial aPCu. Activity in the right thalamus and SMA was also observed.

**Table 3:** Activated brain regions identified for copying a sentence vs. copying a grocery list in MNI coordinate space.

Active region	Hemisphere	*Z*-score	Uncorrected *p*-values	MNI Coordinates			(mm)
SPL	L	6	2.0e-9	-16	-64	60
ITG/MiOG (VWFA)	L	5.8	6.6e-9	-54	-68	-14
ITG/MiOG (VWFA)	R	6	2.0e-9	42	-76	-16
Lingual gyrus	L	5.6	2.1e-8	0	-86	-16
Lingual gyrus	R	5.3	1.2e-7	26	-84	-10
MiFG	L	5.1	3.4e-7	-40	40	34
SMA	R	5.7	1.2e-8	4	18	64
Precuneus	L	5	5.7e-7	-26	-62	52
SuMG	L	4.7	2.6e-6	-62	-26	40
Thalamus	R	4.6	4.2e-6	2	-14	10
IFG	L	4.4	l.le-5	-54	12	38
Posterior cingulate	L	4.4	l.le-5	0	-54	10
Medial aPCU	L	4.8	1.6e-6	-6	-48	70

*IFG, inferior frontal gyrus; MiFG, middle frontal gyrus; SFG, superior frontal gyrus; ITG, inferior temporal gyrus; MiOG, middle occipital gyrus; SPL, superior parietal lobule; SMA, supplementary motor area; PostCB, posterior cerebellum; SuMG, supramarginal gyrus; VWFA, visual word form area; aPCU, anterior precuneus; MNI, Montreal Neurological Institute*.

## Discussion

The present work illustrates the brain activity and behavior of young adults who performed an NP test that was originally designed for assessment of handwriting impairments in patients with AD (Werner et al., 2006). The original test required patients to interact with a digitizing tablet, enabling various kinematic variables to be quantified. These capabilities were mirrored in the present study by incorporating a fMRI-compatible tablet system for handwriting with VFHP, with good ecological validity (Karimpoor et al., 2015). The results supported both hypotheses posited in the Introduction, namely that the observed brain activity (a) consists of a distributed network of regions in reasonable agreement with previous neuropsychological and neuroimaging studies of handwriting performance; and (b) shows differences between the NP test subcomponents consistent with different demands of neural processing for successful task performance. The following discussion evaluates these findings in more detail, focusing first on the behavioral performance and kinematic analysis of the handwriting tasks, and then on the brain activity.

### Behavior

The behavioral performance results of the present study are encouraging, as they replicate some of the observations found by Werner et al. (2006). Werner et al. (2006) observed that across handwriting tasks, the IAT metric was a good discriminator of the differences in handwriting performance across AD, MCI, and healthy control subjects. The IAT metric was also sensitive in the present study, exhibiting a statistically significant decrease when young healthy adults performed the sentence task in comparison to the phone number task and the grocery list task. Similarly, the two studies consistently found that the sentence task was associated with increased writing speed in comparison to the other two tasks, and that all three tasks showed little differences in terms of force production. Beyond this qualitative agreement, however, it is not useful to perform a direct comparison of the absolute metric values reported in both studies for at least three reasons: (1) age differences in the control groups; (2) language differences [English in the present study, Hebrew in Werner et al. (2006)]; and (3) differences in completion time, as the present study involved some task modification to accommodate use of the custom tablet [which has reduced surface area compared to that used by Werner et al. (2006), and to enable a simple block design paradigm followed by group fMRI analysis].

In addition to the qualitative agreement between the two studies, there is a plausible explanation for the dependencies of the handwriting metrics across the three tasks – an issue not addressed by Werner et al. (2006). Compared to the grocery list and phone number tasks, the sentence task was performed with reduced IAT and increased speed. Furthermore, the decrease in IAT occurred even though the sentence task included 13–14 more characters on average in comparison to the other two tasks. The likely reason for these results is that writing a sentence is a more “fluid” task than the other two tasks. Subjects were easily able to read text and then copy it while handwriting from left to right, as in normal everyday activities, moving smoothly from word to word. Both the other tasks are less fluid in the sense that they require copying of random words or numbers that are not assembled in a strong semantic framework, with successive words and phone numbers proceeding from top to bottom, necessitating comparatively large diagonal movements to start each response on a new line. However, it must be emphasized that this interpretation is conjecture at present without support from scientific evidence and literature.

### Brain Activity: All Handwriting Tasks

To our knowledge, no other fMRI study characterized brain activity associated with handwriting using a device similar to that used in the present study, that closely approximates the complex tactile, visual-spatial, and eye-hand coordination elements of real-world performance. Brain activity has previously been reported for writing in the air with the index finger (Katanoda et al., 2001), and writing letters on top of each other on a piece of paper without VFHP (Beeson et al., 2003). Several other studies focusing on language, reading, and spelling aspects of handwriting have involved responses that also have ecological validity limitations (Beeson et al., 2003; Longcamp et al., 2003; Dehaene et al., 2015). Most recently, Planton et al. (2017) also used a tablet for writing during a fMRI study, although without VFHP and without visual feedback of writing production. With “ground truth” lacking, the brain activations observed in the present study are compared with the existing pertinent fMRI literature available on tasks that attempt to engage similar domains of brain function. This provides an initial assessment of consistency, recognizing that additional research will be required in the future to provide stronger evidence and characterization of the neural correlates of this NP test of handwriting performance.

Considering all three writing tasks grouped together vs. baseline, the observed brain activity was consistent with performing a sensorimotor task using the dominant hand for writing and language responses. Predominantly positive *z*-scores were observed in the left lateralized primary somatosensory and motor cortex are consistent with the tactile and proprioceptive sensory inputs used to direct complex hand and arm movements during writing. Activation of pre-central, premotor, SMA, and the VPMc regions is consistent with planning stylus movements required to perform handwriting while visually guiding hand actions. Activation of the SPL is consistent with visual-spatial processing involved in handwriting. In particular, the involvement of the SPL (especially close to the IPL) has been observed in several neuroimaging and lesion studies of writing, and has been referred to as a writing-specific region (Katanoda et al., 2001; Menon and Desmond, 2001; Sugihara et al., 2006; Roux et al., 2009; Segal and Petrides, 2012). In addition, Segal and Petrides (2012) reported the role of the SPL in higher-level motor control involving interactions with language-processing regions during writing performance. Activation of the left angular gyrus and SuMG in the parietal lobe is consistent with the reading and semantic processing aspects of handwriting (Basso et al., 1978; Roeltgen and Heilman, 1984; Segal and Petrides, 2012).

Next, activation of the GMFA located in the MiFG/SFG, is consistent with orthographic processing, processing of graphomotor shapes of words and higher-level motor planning of a complex motor task. The observed activation of the GMFA is consistent with the findings of Planton et al. (2013) who conducted meta-analysis of cerebral network of areas commonly activated during handwriting in 18 neuroimaging studies. The GMFA has been suggested as a writing-specific region in previous fMRI studies (Roux et al., 2009; Segal and Petrides, 2012; Planton et al., 2017). In particular, Roux et al. (2009) conducted direct cortical electrical stimulation of this region and observed impaired handwriting in six patients, without disturbing hand movements or oral language tasks. The GMFA is believed to be involved in bridging between orthography and motor programs specifically during handwriting (Roux et al., 2009). Rapp and Dufor (2011) also suggested that the GMFA is engaged in orthographic-specific working memory during writing.

The observed activation of the IFG and posterior STG is consistent with language processing during handwriting performance (Turkeltaub et al., 2002). Although the NP test under investigation is described above as involving three different copying tasks, a strong language processing component is expected as subjects inevitably engaged in silent reading and comprehension of the stimuli. The left Broca’s area located in IFG is known to be involved in language production and semantic aspects of language processing (Friederici et al., 2003; Winhuisen et al., 2005). Activation of the posterior STG, known as Wernicke’s area, is consistent with language processing, phonetic sequencing and comprehension (Hickok and Poeppel, 2007).

The bilateral involvement of the insula is also consistent with sensory imagery experience and emotional context of writing tasks, as known to be processed by these regions (Craig, 2009). Furthermore, activation of the left MiOG, close to the posterior ITG is referred to as the VWFA and is known to be involved in the storage or recovery of the visual graphic images of words (Nakamura et al., 2000, 2002; Beeson et al., 2003; Rapcsak and Beeson, 2004; Planton et al., 2013).

Lastly, bilateral activity was observed in the cerebellum with involvement of the right posterior region (postCB), as indicated by positive *Z*-scores. Whereas the left cerebellar activation is consistent with representation of finger movements, right cerebellar activation has been similarly observed in previous fMRI studies of writing as approximated by complex, coordinated movement of the index finger, in contrast with simple movements (Katanoda et al., 2001; Segal and Petrides, 2012).

### Brain Activity: Differences between Tasks

Considering first the contrast between copying sentences and copying phone numbers, the observed brain activity was consistent with the differing levels of engagement of language processing areas during complex sentence processing vs. more simple number processing. In particular, the observed activation of left IFG is consistent with more processing requirements for motor and language representation of letters compared to digits. The left IFG is part of the left VPMc and is known to be involved in the storage of motor representation of letters (Longcamp et al., 2003, 2005), or phonological processing (Omura et al., 2004). Involvement of the left SuMG and angular gyrus was also observed, consistent with complex semantic sentence processing vs. comparatively less processing of digits. In addition, activation of the VWFA is consistent with the storage and recovery of the visual graphic images of the words required for processing a sentence, rather than numbers (Nakamura et al., 2000, 2002; Beeson et al., 2003; Rapcsak and Beeson, 2004). The activation of SFG/MiFG (part of the GMFA) suggests preferential involvement of the GMFA during writing letters compared to writing digits, in agreement with the previous findings of Longcamp et al. (2014).

Activation of the left anterior SPL/IPL was also observed, suggesting more engagement of this region associated with the act of handwriting as well as high-level motor control functions required during writing a sentence (Segal and Petrides, 2012; Planton et al., 2017), compared to writing phone numbers, as measured by predominantly positive *Z*-scores in this region. Activation of anterior SPL/IPL and GMFA regions have been consistently associated with writing (Roux et al., 2009; Segal and Petrides, 2012; Planton et al., 2017), highly coordinated fine motor movements and, more recently, drawing (Planton et al., 2013, 2017). Planton et al. (2017) also showed that all regions known as “writing specific” regions were activated during drawing tasks whereas a subset were activated during spelling tasks, thus showing that the specialization of these regions was not absolute but preferential.

Rapp and Dufor (2011) also suggested that activity in these two writing-specific brain regions is modulated by word length (letters) during writing. The contrasted brain activity of the present study is consistent with such an effect, when taken together with the behavioral data observed for the three different handwriting tasks. Specifically, handwriting speed was elevated when copying sentences, in relation to the other two tasks, and thus a greater number of characters were written during the fixed task analysis length of 14 s. However, no firm conclusions can be drawn concerning whether relative involvement of GMFA and SPL/IPL in the current study depends much more on character length, much more on the language and motor requirements for writing words compared to digits, or relatively equal proportions of both mechanisms. This can be considered a limitation of the present study design. Future research to study these mechanisms in detail can be undertaken by designing tasks with appropriate stimulus and control conditions. Lastly for this contrast, the observed involvement of left sensorimotor regions as well as medial aPCu is consistent with use of more sensorimotor and proprioceptive resources (Nachev et al., 2008; Filimon et al., 2009) during copying of a sentence in comparison to copying phone numbers; and with the observed behavioral differences in handwriting metrics (higher writing speed and lower IAT when copying sentences).

Turning to the activation contrasts between copying a sentence and copying a grocery list, differences were again expected due to the different levels of language processing involved for the two tasks (more for copying complex sentences, less for copying a word list). Notably, less regional activity was observed for this contrast in comparison to that for copying sentences vs. copying phone numbers, as the latter contrast involves a larger difference in the level of language processing between the two tasks. In particular, copying sentences vs. copying a grocery list was associated with activation of the left SuMG, and the left MiFG/IFG. This is consistent with the findings of Segal and Petrides (2012), who observed preferential engagement of the left SuMG during writing English words in a strong semantic framework.

Furthermore, copying sentences vs. copying a grocery list showed increased activity in the lingual gyrus and secondary visual areas, SMA, precuneus, and left medial aPCU. This pattern of brain activity is consistent with the significant decrease in IAT values (i.e., more tablet contact time) associated with elevated sensorimotor planning during the former task. The activity of secondary visual areas is known to be associated with processing of complex shapes (Kravitz et al., 2013). The observed bilateral activation of the VWFA is consistent with requirements for storage and recovery of the visual graphic images of words, and as for the contrast discussed immediately above, the word-count was larger for copying sentences. One proviso concerning this interpretation is that the VWFA is known to be associated with reading and visually recognizing shapes of words, but some studies also suggest that the high-order function of this visual processing area is not only limited to reading (Cohen et al., 2000; Dehaene et al., 2005). Planton et al. (2017), observed bilateral activity of the VWFA during drawing that was stronger than the activity during writing, concluding that the activity in this region is vision-related and due to complex object recognition and visual recognition of the words. Additional research is required to provide converging evidence and resolve these differences in interpretation.

## Conclusion

A fMRI study was performed in young healthy adults to identify the neural correlates of an NP test originally designed to assess handwriting impairments in patients with AD. The behavioral findings were consistent with those of Werner et al. (2006) reaffirming that the IAT kinematic metric is sensitive for discriminating performance differences across the three subcomponents of the NP test. The observed brain activity across all tasks showed good consistency with the previous fMRI literature, which to date has involved handwriting tasks implemented with less ecological validity. In particular, activity in the SPL, GMFA, and VWFA are consistent with handwriting specific regions in literature, as well as the involvement of regions in VPMc that are involved in guiding hand actions. Regarding brain activity contrasting the differences between handwriting tasks, the results were consistent with lower, intermediate and higher levels of mental processing for copying phone numbers, items of a grocery list, and sentences, which were further supported by the differences quantified in the behavioral performance data.

These findings are relevant for future fMRI studies that aim to clarify in more detail the functional roles of brain regions that process handwriting behavior. They are also important for providing baseline behavioral and brain activity results in relation to fMRI studies that adopt this handwriting NP test to characterize patients with brain impairments, such as those with AD or prodromal dysfunction, as part of larger testing protocols. The present results are preliminary, given the small sample size, and need to be replicated. Nevertheless, robust analysis procedures have been used to obtain the results and the findings are sufficiently strong to consider using the tablet with VFHP for fMRI of handwriting tasks in AD patients in the near future.

## Ethics Statement

This study was carried out in accordance with the recommendations of ‘Human Research Protections Program, Sunnybrook’s Research Ethics Board (REB)’ with written informed consent from all subjects. All subjects gave written informed consent in accordance with the REB guidelines. The protocol was approved by the ‘Sunnybrook’s research ethics board committee’ (approval number 080-2011).

## Author Contributions

MK: Designing the entire work, data acquisition and experimental setup, data analysis and interpretation; writing and drafting the work; final approval of the work; ensuring questions related to the work accuracy are investigated and resolved. NC: Contributions to the work conception; data analysis, and interpretation of data; revising the work critically; final approval; ensuring questions related to the work accuracy are investigated and resolved. FT: Contributions to the work conception, experimental setup; revising the work critically; final approval of the work; ensuring questions related to the work accuracy are investigated and resolved. CF: Contributions to the work conception; critically revising the work; final approval of the work; ensuring questions related to the work accuracy are investigated and resolved. TS: Contributions to the work conception and data interpretation; revising the work critically; final approval of the work; ensuring questions related to the work accuracy are investigated and resolved. SG: Designing the work; analysis and interpretation of data; revising the work critically; final approval of the work; ensuring questions related to the work accuracy are investigated and resolved.

## Conflict of Interest Statement

This work has used a technology which was provisionally patented in 2014. Patent title: Systems and Methods for Providing Visual Feedback of Touch Panel Input during Magnetic Resonance Imaging. Publication Number: US 2016/0120437 A1. The authors declare that the research was conducted in the absence of any commercial or financial relationships that could be construed as a potential conflict of interest.

## Abbreviations

AC: anterior cingulate
AFNI: analysis of functional neuroimaging
aPCu: anterior precuneus
BOLD: blood oxygen level dependent
FMRI: functional magnetic resonance imaging
FOV: field of view
FWHM: full width half maximum
GMFA: graphomotor frontal area
IAT: in air time
IFG: inferior frontal gyrus
IOG: inferior occipital gyrus
IPL: inferior parietal lobule
ITG: inferior temporal gyrus
LDA: linear discriminant analysis
MeFG: medial frontal gyrus
MiFG: middle frontal gyrus
MiOG: middle occipital gyrus
MiTG: middle temporal gyrus
MNI: Montreal Neurological Institute
PCA: principal component analysis
PostCB: posterior cerebellum
SFG: superior frontal gyrus
SMA: supplementary motor area
SOG: superior occipital gyrus
SPL: superior parietal lobule
SPMs: statistical parametric map
STG: superior temporal gyrus
SuMG: Supramarginal gyrus
VFHP: visual feedback of hand position
VPMc: ventral premotor cortex
VWFA: visual word form area.

## References

[B1] Auerbach S. H. Alexander M. P. (1981). Pure agraphia and unilateral optic ataxia associated with a left superior parietal lobule lesion. *J. Neurol. Neurosurg. Psychiatry* 44 430–432. 10.1136/jnnp.44.5.430 7264690 PMC490988

[B2] Basso A. Taborelli A. Vignolo L. A. (1978). Dissociated disorders of speaking and writing in aphasia. *J. Neurol. Neurosurg. Psychiatry* 41 556–563. 10.1136/jnnp.41.6.556 671067 PMC493084

[B3] Beeson P. Rapcsak S. Plante E. Chargualaf J. Chung A. Johnson S. (2003). The neural substrates of writing: A functional magnetic resonance imaging study. *Aphasiology* 17 647–665. 10.1080/02687030344000067

[B4] Benson D. F. Cummings J. L. Tsai S. Y. (1982). Angular gyrus syndrome simulating Alzheimer’s Disease. *Arch. Neurol.* 39 616–620. 10.1001/archneur.1982.00510220014003 7125973

[B5] Blackwell A. D. Sahakian B. J. Vesey R. Semple J. M. Robbins T. W. Hodges J. R. (2003). Detecting dementia: novel neuropsychological markers of preclinical Alzheimer’s disease. *Dement. Geriatr. Cogn. Disord.* 17 42–48. 10.1159/000074081 14560064

[B6] Carbonell F. Bellec P. Shmuel A. (2011). Global and system-specific resting-state fMRI fluctuations are uncorrelated: principal component analysis reveals anti-correlated networks. *Brain Connect.* 1 496–510. 10.1089/brain.2011.0065 22444074 PMC3604782

[B7] Churchill N. Spring R. Abdi H. Kovacevic N. McIntosh A. R. Strother S. (2013). “The stability of behavioral PLS results in ill-posed neuroimaging problems,” in *New Perspectives in Partial Least Squares and Related Methods*, eds Abdi H. Chin W. Vinzi E. Russolillo G. Trinchera L. (New York, NY: Springer), 171–183.

[B8] Churchill N. W. Oder A. Abdi H. Tam F. Lee W. Thomas C. (2012a). Optimizing preprocessing and analysis pipelines for single-subject fMRI. I. Standard temporal motion and physiological noise correction methods. *Hum. Brain Mapp.* 33 609–627. 10.1002/hbm.21238 21455942 PMC4898950

[B9] Churchill N. W. Spring R. Afshin-Pour B. Dong F. Strother S. C. (2015). An automated, adaptive framework for optimizing preprocessing pipelines in task-based functional MRI. *PLOS ONE* 10:e0131520. 10.1371/journal.pone.0131520 26161667 PMC4498698

[B10] Churchill N. W. Yourganov G. Oder A. Tam F. Graham S. J. Strother S. C. (2012b). Optimizing preprocessing and analysis pipelines for single-subject fMRI: 2. Interactions with ICA, PCA, task contrast and inter-subject heterogeneity. *PLOS ONE* 7:e31147. 10.1371/journal.pone.0031147 22383999 PMC3288007

[B11] Churchill N. W. Yourganov G. Strother S. C. (2014). Comparing within-subject classification and regularization methods in fMRI for large and small sample sizes. *Hum. Brain Mapp.* 35 4499–4517. 10.1002/hbm.22490 24639383 PMC6869036

[B12] Cohen L. Dehaene S. Naccache L. Lehéricy S. Dehaene-Lambertz G. Hénaff M. A. (2000). The visual word form area. *Brain* 123 291–307. 10.1093/brain/123.2.291 10648437

[B13] Cox R. W. (1996). AFNI: software for analysis and visualization of functional magnetic resonance neuroimages. *Comput. Biomed. Res.* 29 162–173. 10.1006/cbmr.1996.0014 8812068

[B14] Craig A. D. (2009). How do you feel—now? the anterior insula and human awareness. *Nat. Rev. Neurosci.* 10 59–70. 10.1038/nrn2555 19096369

[B15] Croisile B. (1999). Agraphia in Alzheimer’s disease. *Dement. Geriatr. Cogn. Disord.* 10 226–230. 10.1159/000017124 10325451

[B16] Dehaene S. Cohen L. Morais J. Kolinsky R. (2015). Illiterate to literate: behavioural and cerebral changes induced by reading acquisition. *Nat. Rev. Neurosci.* 16 234–244. 10.1038/nrn3924 25783611

[B17] Dehaene S. Cohen L. Sigman M. Vinckier F. (2005). The neural code for written words: a proposal. *Trends Cogn. Sci.* 9 335–341. 10.1016/j.tics.2005.05.004 15951224

[B18] Eklund A. Nichols T. E. Knutsson H. (2016). Cluster failure: Why fMRI inferences for spatial extent have inflated false-positive rates. *Proc. Natl. Acad. Sci.* 113 7900–7905. 10.1073/pnas.1602413113 27357684 PMC4948312

[B19] Filimon F. Nelson J. D. Huang R. Sereno M. I. (2009). Multiple parietal reach regions in humans: cortical representations for visual and proprioceptive feedback during on-line reaching. *J. Neurosci.* 29 2961–2971. 10.1523/JNEUROSCI.3211-08.2009 19261891 PMC3407568

[B20] Folstein M. F. Folstein S. E. McHugh P. R. (1975). “Mini-mental state”: a practical method for grading the cognitive state of patients for the clinician. *J. Psychiatr. Res.* 12 189–198. 10.1016/0022-3956(75)90026-6 1202204

[B21] Friederici A. D. Rueschemeyer S. A. Hahne A. Fiebach C. J. (2003). The role of left inferior frontal and superior temporal cortex in sentence comprehension: localizing syntactic and semantic processes. *Cereb. Cortex* 13 170–177. 10.1093/cercor/13.2.170 12507948

[B22] Genovese C. R. Lazar N. A. Nichols T. (2002). Thresholding of statistical maps in functional neuroimaging using the false discovery rate. *Neuroimage* 15 870–878. 10.1006/nimg.2001.1037 11906227

[B23] Ghilardi M. F. Alberoni M. Marelli S. Rossi M. Franceschi M. Ghez C. (1999). Impaired movement control in Alzheimer’s disease. *Neurosci. Lett.* 260 45–48. 10.1016/S0304-3940(98)00957-4 10027696

[B24] Ghilardi M. F. Alberoni M. Rossi M. Franceschi M. Mariani C. Fazio F. (2000). Visual feedback has differential effects on reaching movements in Parkinson’s and Alzheimer’s disease. *Brain Res.* 876 112–123. 10.1016/S0006-8993(00)02635-4 10973599

[B25] Glover G. H. Law C. S. (2001). Spiral-in/out BOLD fMRI for increased SNR and reduced susceptibility artifacts. *Magn. Reson. Med.* 46 515–522. 10.1002/mrm.1222 11550244

[B26] Glover G. H. Li T. Q. Ress D. (2000). Image-based method for retrospective correction of physiological motion effects in fMRI: RETROICOR. *Magn. Reson. Med.* 44 162–167. 10.1002/1522-2594(200007)44:1<162::AID-MRM23>3.0.CO;2-E 10893535

[B27] Halligan P. W. Hunt M. Marshall J. C. Wade D. T. (1996). When seeing is feeling: acquired synaesthesia or phantom touch? *Neurocase* 2 21–29. 10.1080/13554799608402385

[B28] Hebben N. Milberg W. (2009). *Introduction to Neuropsychological Assessment, and Essentials of Interpretation in Essentials of Neuropsychological Assessment*, 2nd Edn Edison, NJ: John Wiley & Sons, 1–25.

[B29] Hickok G. Poeppel D. (2007). The cortical organization of speech processing. *Nat. Rev. Neurosci.* 8 393–402. 10.1038/nrn2113 17431404

[B30] Karimpoor M. Tam F. Strother S. C. Fischer C. E. Schweizer T. A. Graham S. J. (2015). A computerized tablet with visual feedback of hand position for functional magnetic resonance imaging. *Front. Hum. Neurosci.* 9:150. 10.3389/fnhum.2015.00150 25859201 PMC4373274

[B31] Katanoda K. Yoshikawa K. Sugishita M. (2001). A functional MRI study on the neural substrates for writing. *Hum. Brain Mapp.* 13 34–42. 10.1002/hbm.1023 11284045 PMC6871921

[B32] Kovac J. Peer P. Solina F. (2003). *Human Skin Color Clustering for Face Detection*, Vol. 2 Piscataway, NJ: IEEE, 144–148 10.1109/EURCON.2003.1248169

[B33] Kravitz D. J. Saleem K. S. Baker C. I. Ungerleider L. G. Mishkin M. (2013). The ventral visual pathway: an expanded neural framework for the processing of object quality. *Trends Cogn. Sci.* 17 26–49. 10.1016/j.tics.2012.10.011 23265839 PMC3532569

[B34] LaBarge E. Smith D. S. Dick L. Storandt M. (1992). Agraphia in dementia of the Alzheimer type. *Arch. Neurol.* 49 1151–1156. 10.1001/archneur.1992.00530350065021 1444882

[B35] Lezak M. D. (2004). *Neuropsychological Assessment.* New York, NY: Oxford University Press.

[B36] Longcamp M. Anton J. L. Roth M. Velay J. L. (2003). Visual presentation of single letters activates a premotor area involved in writing. *Neuroimage* 19 1492–1500. 10.1016/S1053-8119(03)00088-0 12948705

[B37] Longcamp M. Anton J. L. Roth M. Velay J. L. (2005). Premotor activations in response to visually presented single letters depend on the hand used to write: a study on left-handers. *Neuropsychologia* 43 1801–1809. 10.1016/j.neuropsychologia.2005.01.020 16154456

[B38] Longcamp M. Lagarrigue A. Nazarian B. Roth M. Anton J. L. Alario F. X. (2014). Functional specificity in the motor system: evidence from coupled fMRI and kinematic recordings during letter and digit writing. *Hum. Brain Mapp.* 35 6077–6087. 10.1002/hbm.22606 25093278 PMC6868974

[B39] Maurer K. Volk S. Gerbaldo H. (1997). Auguste D and Alzheimer’s disease. *Lancet* 349 1546–1549. 10.1016/S0140-6736(96)10203-8 9167474

[B40] Mazziotta J. Toga A. Evans A. Fox P. Lancaster J. Zilles K. (2001). A probabilistic atlas and reference system for the human brain: international consortium for brain mapping (ICBM). *Philos. Trans. R. Soc. Lond. B Biol. Sci.* 356 1293–1322. 10.1098/rstb.2001.0915 11545704 PMC1088516

[B41] Menon V. Desmond J. E. (2001). Left superior parietal cortex involvement in writing: integrating fMRI with lesion evidence. *Brain Res. Cogn. Brain Res.* 12 337–340. 10.1016/S0926-6410(01)00063-5 11587904

[B42] Nachev P. Kennard C. Husain M. (2008). Functional role of the supplementary and pre-supplementary motor areas. *Nat. Rev. Neurosci.* 9 856–869. 10.1038/nrn2478 18843271

[B43] Nakamura K. Honda M. Hirano S. Oga T. Sawamoto N. Hanakawa T. (2002). Modulation of the visual word retrieval system in writing: a functional MRI study on the Japanese orthographies. *J. Cogn. Neurosci.* 14 104–115. 10.1162/089892902317205366 11798391

[B44] Nakamura K. Honda M. Okada T. Hanakawa T. Toma K. Fukuyama H. (2000). Participation of the left posterior inferior temporal cortex in writing and mental recall of kanji orthography. *Brain* 123 954–967. 10.1093/brain/123.5.954 10775540

[B45] Nichols T. E. Hayasaka S. (2003). Controlling the familywise error rate in functional neuroimaging: a comparative review. *Stat. Methods Med. Res.* 12 419–446. 10.1191/0962280203sm341ra 14599004

[B46] Ogawa S. Lee T. M. Kay A. R. Tank D. W. (1990). Brain magnetic resonance imaging with contrast dependent on blood oxygenation. *Proc. Natl. Acad. Sci. U.S.A.* 87 9868–9872. 10.1073/pnas.87.24.9868 2124706 PMC55275

[B47] Ogawa S. Tank D. W. Menon R. Ellermann J. M. Kim S. G. Merkle H. (1992). Intrinsic signal changes accompanying sensory stimulation: functional brain mapping with magnetic resonance imaging. *Proc. Natl. Acad. Sci. U.S.A.* 89 5951–5955. 10.1073/pnas.89.13.5951 1631079 PMC402116

[B48] Oldfield R. C. (1971). The assessment and analysis of handedness: the Edinburgh inventory. *Neuropsychologia* 9 97–113. 10.1016/0028-3932(71)90067-4 5146491

[B49] Omura K. Tsukamoto T. Kotani Y. Ohgami Y. Yoshikawa K. (2004). Neural correlates of phoneme-to-grapheme conversion. *Neuroreport* 15 949–953. 10.1097/00001756-200404290-00004 15076713

[B50] Otsuki M. Soma Y. Arai T. Otsuka A. Tsuji S. (1999). Pure apraxic agraphia with abnormal writing stroke sequences: report of a Japanese patient with a left superior parietal haemorrhage. *J. Neurol. Neurosurg. Psychiatry* 66 233–237. 10.1136/jnnp.66.2.233 10071107 PMC1736213

[B51] Planton S. Jucla M. Roux F. E. Démonet J. F. (2013). The “handwriting brain”: a meta-analysis of neuroimaging studies of motor versus orthographic processes. *Cortex* 49 2772–2787. 10.1016/j.cortex.2013.05.011 23831432

[B52] Planton S. Longcamp M. Péran P. Démonet J. F. Jucla M. (2017). How specialized are *writing-specific* brain regions? An fMRI study of writing, drawing and oral spelling. *Cortex* 88 66–80. 10.1016/j.cortex.2016.11.018 28081451

[B53] Platel H. Lambert J. Eustache F. Cadet B. Dary M. Viader F. (1993). Characteristics and evolution of writing impairmant in Alzheimer’s disease. *Neuropsychologia* 31 1147–1158. 10.1016/0028-3932(93)90064-7 8107977

[B54] Rapcsak S. Z. Beeson P. M. (2004). The role of left posterior inferior temporal cortex in spelling. *Neurology* 62 2221–2229. 10.1212/01.WNL.0000130169.60752.C5 15210886

[B55] Rapp B. Dufor O. (2011). The neurotopography of written word production: an fMRI investigation of the distribution of sensitivity to length and frequency. *J. Cogn. Neurosci.* 23 4067–4081. 10.1162/jocn_a_00109 21812571

[B56] Rasmussen P. M. Hansen L. K. Madsen K. H. Churchill N. W. Strother S. C. (2012). Model sparsity and brain pattern interpretation of classification models in neuroimaging. *Pattern Recognit.* 45 2085–2100. 10.1016/j.patcog.2011.09.011

[B57] Reisman J. E. (1993). Development and reliability of the research version of the minnesota handwriting test. *Phys. Occupat. Ther. Pediatr.* 13 41–55. 10.1080/J006v13n02_03

[B58] Roeltgen D. P. Heilman K. M. (1984). Lexical agraphia. *Brain* 107 811–827. 10.1093/brain/107.3.811 6206909

[B59] Rosenblum S. Weiss P. L. Parush S. (2003). Product and process evaluation of handwriting difficulties. *Educ. Psychol. Rev.* 15 41–81. 10.1023/A:1021371425220 16541982

[B60] Roux F.-E. Dufor O. Giussani C. Wamain Y. Draper L. Longcamp M. (2009). The graphemic/motor frontal area Exner’s area revisited. *Ann. Neurol.* 66 537–545. 10.1002/ana.21804 19847902

[B61] Schröter A. Mergl R. Bürger K. Hampel H. Möller H. J. Hegerl U. (2003). Kinematic analysis of handwriting movements in patients with Alzheimer’s disease, mild cognitive impairment, depression and healthy subjects. *Dement. Geriatr. Cogn. Disord.* 15 132–142. 10.1159/000068484 12584428

[B62] Segal E. Petrides M. (2012). The anterior superior parietal lobule and its interactions with language and motor areas during writing. *Eur. J. Neurosci.* 35 309–322. 10.1111/j.1460-9568.2011.07937.x 22188383

[B63] Slavin M. J. Phillips J. G. Bradshaw J. L. Hall K. A. Presnell I. (1999). Consistency of handwriting movements in dementia of the Alzheimer’s type: a comparison with Huntington’s and Parkinson’s diseases. *J. Int. Neuropsychol. Soc.* 5 20–25. 10.1017/S135561779951103X 9989020

[B64] Smith S. M. Jenkinson M. Woolrich M. W. Beckmann C. F. Behrens T. E. Johansen-Berg H. (2004). Advances in functional and structural MR image analysis and implementation as FSL. *Neuroimage* 23 S208–S219. 10.1016/j.neuroimage.2004.07.051 15501092

[B65] Strauss E. Sherman E. M. Spreen O. (2006). *A Compendium of Neuropsychological Tests: Administration, Norms, and Commentary.* Washington, DC: American Chemical Society.

[B66] Strother S. La Conte S. Hansen L. K. Anderson J. Zhang J. Pulapura S. (2004). Optimizing the fMRI data-processing pipeline using prediction and reproducibility performance metrics: I. A preliminary group analysis. *Neuroimage* 23 S196–S207. 15501090 10.1016/j.neuroimage.2004.07.022

[B67] Strother S. C. Anderson J. Hansen L. K. Kjems U. Kustra R. Sidtis J. (2002). The quantitative evaluation of functional neuroimaging experiments: the NPAIRS data analysis framework. *Neuroimage* 15 747–771. 10.1006/nimg.2001.1034 11906218

[B68] Sugihara G. Kaminaga T. Sugishita M. (2006). Interindividual uniformity and variety of the ‘Writing center’: a functional MRI study. *Neuroimage* 32 1837–1849. 10.1016/j.neuroimage.2006.05.035 16872841

[B69] Tam F. Churchill N. W. Strother S. C. Graham S. J. (2011). A new tablet for writing and drawing during functional MRI. *Hum. Brain Mapp.* 32 240–248. 10.1002/hbm.21013 20336688 PMC6870006

[B70] Tigges P. Mergl R. Frodl T. Meisenzahl E. M. Gallinat J. Schröter A. (2000). Digitized analysis of abnormal hand–motor performance in schizophrenic patients. *Schizophr. Res.* 45 133–143. 10.1016/S0920-9964(99)00185-1 10978881

[B71] Turkeltaub P. E. Eden G. F. Jones K. M. Zeffiro T. A. (2002). Meta-analysis of the functional neuroanatomy of single-word reading: method and validation. *Neuroimage* 16 765–780. 10.1006/nimg.2002.1131 12169260

[B72] Werner P. Rosenblum S. Bar-On G. Heinik J. Korczyn A. (2006). Handwriting process variables discriminating mild Alzheimer’s disease and mild cognitive impairment. *J. Gerontol. Ser. B Psychol. Sci. Soc. Sci.* 61 228–236. 16855035 10.1093/geronb/61.4.p228

[B73] Winhuisen L. Thiel A. Schumacher B. Kessler J. Rudolf J. Haupt W. F. (2005). Role of the contralateral inferior frontal gyrus in recovery of language function in poststroke aphasia A combined repetitive transcranial magnetic stimulation and positron emission tomography study. *Stroke* 36 1759–1763. 10.1161/01.STR.0000174487.81126.ef 16020770

[B74] Yokota T. Ishiai S. Furukawa T. Tsukagoshi H. (1990). Pure agraphia of kanji due to thrombosis of the Labbe vein. *J. Neurol. Neurosurg. Psychiatry* 53 335–338. 10.1136/jnnp.53.4.335 2341848 PMC1014173

[B75] Yorkston K. M. Jaffe K. M. Polissar N. L. Liao S. Fay G. C. (1997). Written language production and neuropsychological function in children with traumatic brain injury. *Arch. Phys. Med. Rehabil.* 78 1096–1102. 10.1016/S0003-9993(97)90134-9 9339159

[B76] Yourganov G. Schmah T. Churchill N. W. Berman M. G. Grady C. L. Strother S. C. (2014). Pattern classification of fMRI data: applications for analysis of spatially distributed cortical networks. *Neuroimage* 96 117–132. 10.1016/j.neuroimage.2014.03.074 24705202

